# Carbon Nanotube Field-Effect Transistor-Based Chemical and Biological Sensors

**DOI:** 10.3390/s21030995

**Published:** 2021-02-02

**Authors:** Xuesong Yao, Yalei Zhang, Wanlin Jin, Youfan Hu, Yue Cui

**Affiliations:** 1 School of Materials Science and Engineering, Peking University, Beijing 100871, China; 1801214055@pku.edu.cn (X.Y.); zhangyalei@pku.edu.cn (Y.Z.); 2 Key Laboratory for the Physics and Chemistry of Nanodevices, Center for Carbon-Based Electronics, Frontiers Science Center for Nano-Optoelectronics, and Department of Electronics, Peking University, Beijing 100871, China; 1801213549@pku.edu.cn

**Keywords:** carbon nanotube, field-effect transistors, sensors, chemical, biological

## Abstract

Chemical and biological sensors have attracted great interest due to their importance in applications of healthcare, food quality monitoring, environmental monitoring, etc. Carbon nanotube (CNT)-based field-effect transistors (FETs) are novel sensing device configurations and are very promising for their potential to drive many technological advancements in this field due to the extraordinary electrical properties of CNTs. This review focuses on the implementation of CNT-based FETs (CNTFETs) in chemical and biological sensors. It begins with the introduction of properties, and surface functionalization of CNTs for sensing. Then, configurations and sensing mechanisms for CNT FETs are introduced. Next, recent progresses of CNTFET-based chemical sensors, and biological sensors are summarized. Finally, we end the review with an overview about the current application status and the remaining challenges for the CNTFET-based chemical and biological sensors.

## 1. Introduction

Chemical and biological sensors have attracted great attention recently, and have a wide range of applications in healthcare, environmental monitoring, food quality, and defense. These sensors can respond to specific chemical or biological compounds and convert this information into electrical signals. Many materials have been studied as the sensitive materials in the chemical/biological sensors, such as SnO_2_ [1,2], ZnO_2_ [3], Ag [4], and graphene [5]. Generally speaking, the ideal material in chemical and biological sensors should have a high chemical reactivity, a large surface to volume ratio or an easy fabrication at low cost. 

Carbon nanotubes (CNTs) are seamless nanotubes made of single or multiple layers of graphene sheets rolled around a central axis with the advantages of being lightweight and having a perfect hexagonal connection structure. The unique electronic transport properties of CNTs make them potentially useful in nanodevices [6,7]. For example, CNTs are atomically thin in order to provide ideal electrostatic control over the channel, which is quite important when the device is scaled down. This unique atomically thin structure of CNTs also gives them many advantages when serving as the sensitive materials in sensors, and the electrical performance superiority of CNT-based field effect transistors (CNTFETs) has been extended in various chemical and biological sensors. Compared with other detecting technologies, CNTFET-based sensors have the advantages of high sensitivity, high selectivity, simple operation, low operating temperature, fast response speed, short recovery time, label-free detection, and good stability. Referring to the detection capability of various substances and the exceptional performance, CNTFETs are expected to play an increasing role in the field of sensing. In this review, the properties of CNTs, configurations and sensing mechanisms based on CNTFETs, recent progresses of the implementation of CNTFETs in chemical sensors and biological sensors, and the perspective of this technology are introduced. 

## 2. Surface Functionalization of CNTs for Sensing

### 2.1. Covaelent Modification

**Figure 1:**
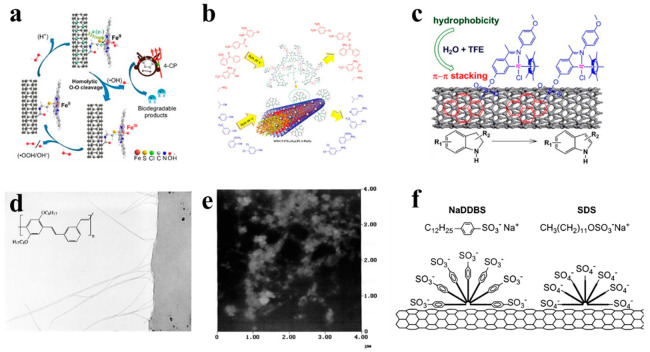
Surface functionalization of carbon nanotubes (CNTs). (**a**) The introduction of the -COOH group to the surface of the CNT [8]. (**b**) The introduction of-COOH on the surface of CNTs by the oxidation reaction [9]. (**c**) A metal iridium complex catalyst was coated on the surface of CNTs through non-covalent bond accumulation [10]. (**d**) TEM image which shows the nominal chemical structure of the polymer backbone [11]. (**e**) The atomic force microscopy image of the waxy corn amylopectin-single-walled CNT (SWCNT) film [12]. (**f**) Schematic representation of surfactants adsorb onto the CNT surfaces [13].

Covalent modification mainly involves chemical destruction of C-C bonds of the CNTs ports or sidewalls to generate more polar carboxyl groups or hydroxyl groups on the surfaces. Then, various functional groups can be introduced on the CNT surfaces to further attach the derivative reaction of the target product to the CNTs—for example, chemical groups, fluorescently labeled molecules, DNA, anticancer drugs, etc. The oxidants used for covalently modification include nitric acid, mixed acid (concentrated sulfuric acid/nitric acid), neutral hydrogen peroxide, and sodium hydroxide, as shown in Figure 1. The disadvantage of covalent modification is that it may destroy the integrity of CNTs, which will affect its mechanical and electrical properties to some extent. 

As shown in Figure 1a, Ni et al. [8] introduced the -COOH derivative group on the surface of the CNT by the oxidation reaction, and two different ligands are introduced on the CNT surface, including aminopyridine and aminoethyl mercaptan curing catalyst iron phthalocyanine (VE-H). As shown in Figure 1b, Rezaie et al. [9] also introduced -COOH to the surface of CNTs by oxidation reaction, then formed a dendrimer with poly(citric acid), and then attached a divalent platinum metal catalyst to the surface of CNTs. Finally, a magnetic catalyst was obtained. The catalyst can selectively reduce nitro and nitrile.

### 2.2. Non-Covalent Modification

Non-covalent modification means that covalent chemical bonds are not introduced for modification on the surface of CNTs, but are achieved through non-covalent bonding, including physical adsorption and surface coating. The non-covalent modification is mainly formed by the hybridization of carbon atoms sp^2^ in the graphene structure of the sidewall to form highly delocalized electrons and electrons of other compounds to generate non-covalent bonds. The non-covalent interactions include dispersion forces, hydrogen bonds, dipole-dipole forces, π-π stacking effects, and hydrophobic effects. Carbon atoms in CNTs are all SP^2^ hybrids, forming highly delocalized electrons, which can be modified with other π electron-rich compounds through π-π stacking. The non-covalently modified CNTs are structurally complete and can retain their original properties. The molecules for the non-covalent modification mainly include the surfactants, the molecules containing aromatic groups, and the polymers. For biological sensors, the non-covalent modification of the CNTs can not only improve their water solubility in biological systems, but also can avoid the non-specific adsorption of the biomolecules.

As shown in Figure 1c, Liu et al. [10] coated the metal iridium complex catalyst on the surface of CNTs through non-covalent bond accumulation, and the coating efficiency reached over 94%. The metal iridium complex catalyst is coated on CNT to make the catalytic dehydrogenation reaction of indole from organics such as methanol, ethanol, tetrahydrofuran, trifluoroethanol, etc. The transfer of solvents to water makes this organic reaction more environmentally friendly. The surfactant contains two parts, which are the lipophilic end and the hydrophilic end. When they are adsorbed on the surface by the CNTs, the charge repulsion disperses them. Under thermodynamics, water-soluble polymers such as sodium polystyrene sulfonate entangle CNTs, thereby exerting the role of surfactants and making them amphiphilic [14]. As shown in Figure 1d, Dalton et al. [11] used conjugated poly-phenylene vinylene to surface-coat the CNTs, and found that the CNTs were evenly dispersed in the polymer matrix. As shown in Figure 1e, Stobinski et al. [12] modified CNTs with sodium lauryl sulfate and dispersed them by ultrasound. It was found that increasing the ultrasonic time or decreasing the concentration of the suspension can obtain CNT solutions with better dispersion properties. As shown in Figure 1f, coating CNTs with sodium benzoate and sodium dodecyl benzenesulfonate can also increase their water solubility [13].

## 3. Configuration and Sensing Mechanism

### 3.1. General CNTFETs 

CNTFETs have a variety of structures [15], but share similar characteristics: the conductive channel, source and drain electrodes, a gate electrode on the top or bottom of the channel, and a dielectric layer between the channel and the gate to separate the gate electrode from the CNTs. The operating principles of these CNTFETs are similar: the gate electrode uses a vertical electric field to control the amount of charge in the channel; the horizontal electric field between the source and drain electrodes provides driving force, and a current is made to flow from one electrode through the CNT to the other electrode [16].

**Figure 2:**
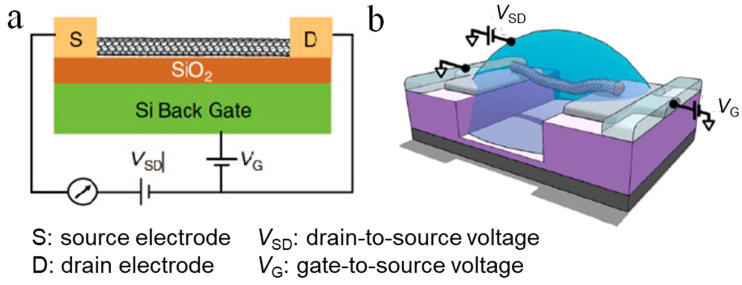
Illustration of CNT-based field-effect transistors (FETs) for sensing. (**a**) Schematic representation of a general CNTFET [17]. (**b**) Schematic diagram of an electrolyte-gated CNTFET [20].

Figure 2a shows a typical configuration of a CNTFET for sensing purpose. In general, the transport of carriers in a CNTFET can be attributed to four states, which are independent of the device structure [17]. The classification of these states depends on the comparison of the CNT length and the mean free path length of the CNT, and the type of contact between the CNT and the source and drain [18]. For example, an ohmic contact ballistic CNTFET means that carriers are injected into the CNTs from the source and the drain through an ohmic contact, and the carrier transport process in the CNT is not subjected to any scattering. In contrast, Schottky-type diffused CNTFETs mean that the carrier injection is affected by a Schottky barrier derived from the heterojunction of the electrode and the CNT, and the carriers are constantly scattered during transmission in the conductive channel. There are two kinds of carriers: holes and electrons. If the type of carriers is mainly electrons, then the FET is an n-type transistor; on the other hand, if the carriers are mainly holes, then the FET is a p-type transistor. In theory, the type of metal-CNT contact depends on the difference in work function between the metal electrode and the CNTs. However, due to the physical and chemical properties of the electrodes in contact with CNTs [19], p-type CNTFETs are more common. 

CNTs have the characteristics of nanometer size, huge specific surface area, and surface effects. When a specific molecule is adsorbed on the surface of a CNTs, it causes the energy band of the CNTs to bend and affect its electronic structure, which would further cause the change of transport characteristics of CNTs. The change provides the possibility of CNTs to work as sensitive materials. 

The FET-based chemical sensor and biosensor use the basic characteristics of the transistor to convert difficult-to-detect high-resistance changes into easily-detectable changes in current. The sensitivity of the sensor can be adjusted by appropriately selecting the gate operating voltage of the device. The single-walled CNTs (SWCNTs) can be divided into metal type and semiconductor type, and the FET-based chemical sensor and biosensor are prepared by using the resistance response characteristic of semiconductor SWCNTs to the adsorbed chemical.

When the chemical molecules or biological material are adsorbed on the surface of semiconductor-type CNTs, electron transfer occurs, which changes their electrical conductivity, which provides a theoretical basis for CNTs as good sensor materials. The multi-walled CNTs (MWCNTs) have a multi-layered tubular structure, so the MWCNTs have a more complex chemical adsorption mechanism than the SWCNTs. In addition, the MWCNTs lack carbon band gaps or have narrow band gaps; the tube is mainly metallic so that the adsorption of the chemical molecules has little effect on the electrical conductivity. Therefore, the conductivity of the MWCNTs is not as sensitive as that of SWCNTs, but it has been shown that the MWCNTs still have excellent sensing characteristics to substances such as water vapor [21], NH_3_ [22,23], NO_2_ [18], and O_2_ [24,25].

The SWCNTs have shown great advantages in FET-based nanosensors. Paolo et al. [26] reported the application of CNTFETs in gas sensors. Whether for a single CNT FET or a CNT-thin film FET, the main sensing mechanism is derived from gas adsorption on the carbon tubes/metals—the modulation of the Schottky barrier at the electrodes. 

### 3.2. Electrolyte-Gated CNTFETs

The biosensors based on electrolyte-gated FETs, also known as liquid gate FETs, have attracted increasing attention due to their advantages of easy processing [27], low cost [28], good flexibility [29], good biocompatibility, and low operating voltage. Figure 2b shows a typical configuration of a electrolyte-gated CNTFET [20], in which the electrolyte is used instead of the dielectric layer material to directly contact the gate electrode and the channel. The biggest difference between the working principle of the electrolyte-gated FET and the conventional FET is that the gate electrode regulates the channel current through the electrolyte solution. The biggest advantage of electrolyte-gated FETs is the huge two-electron layer effect of the electrolyte [30,31], which enables the sensor to obtain the same current with a smaller gate voltage and usually can work at a rather low voltage (1V). This can avoid undesired electrochemical reactions, such as decomposition of water, damage to biological activity, etc.; thus, it can be used to detect important biological samples in a solution environment [32,33].

According to whether the ions in the electrolyte can penetrate the semiconductor channel layer, the electrolyte-gated FET can be divided into an electrostatically coupled FET and an electrochemically doped FET. Taken the p-type electrolyte-gated FET as an example, we explain these two different working mechanisms as follows. (1) Electrostatically coupled FETs. Under negative gate bias, cations in the electrolyte migrate to the gate/electrolyte interface, while the anions move to the electrolyte/channel interface, and an electric double layer is formed at the respective interface. The capacitance of the entire sensor can be equivalent to two electric double-layer capacitors connected in series. Usually, the capacitance at the electrolyte/channel interface is small, so this capacitance determines the total capacitance. Under negative bias, anions accumulated at the electrolyte/channel interface in the electrolyte induce holes of equal charge amount in the p-type channel. Under the action of the source-drain voltage, holes move in the channel to form a source-drain current. In this process, the anions in the electrolyte are always at the electrolyte/channel interface and do not penetrate the semiconductor channel layer. The holes in the channel are completely generated by the electrostatic coupling of the electric double-layer capacitance. (2) Electrochemically doped FETs. Under negative bias, cations in the electrolyte migrate to the gate/electrolyte interface, while anions migrate to the electrolyte/channel and penetrate the interface into the semiconductor channel layer. The entering anions would cancel or compensate some of the holes. This process is called electrochemical doping and occurs mostly at the electrolyte–polymer semiconductor interface. Both these two methods are often used in CNTFETs.

According to different analytes, the application of electrolyte-gated CNTFETs in chemical and biological sensors mainly includes ion sensors, small molecule sensors, protein sensors, DNA sensors, bacterial sensors, cell sensors, etc. Because the electrolyte-gated FET sensor works in a solution environment, it is particularly important to investigate the interaction between ions and CNTs. According to the interaction between ions and CNTs, ion redox, chloride ion detection, nucleic acid aptamers, and ion-selective membranes can be used for electrolyte-gated CNTFET for ion detection. For example, electrolyte-gated FET with single-walled CNTs could be used to detect redox ions [34]. It was found that ions with redox ability affect the conductivity of CNT channels mainly by adjusting the electrochemical potential of the solution. Boussaad et al. [35] prepared electrolyte-gated CNTFET sensors and studied their response to K_3_Fe(CN)_6_/K_4_Fe(CN)_6_, K_2_IrCl_6_/K_3_IrCl_6_. It was found that redox ions can not only regulate the electrochemical potential of the solution, but also directly conduct a redox reaction with the CNT, thereby regulating the conductivity of the CNT channel. 

## 4. CNTFET-Based Chemical Sensors

### 4.1. Gas Sensors

The CNTFET-based sensor can provide a high specific surface area for gas adsorption because almost all atoms are exposed to the gas environment, which is helpful in improving the response sensitivity. No matter as a single CNT FET or a CNT thin film FET, the main sensing mechanism is derived from gas adsorption on CNTs/metals. Unlike polycrystal line materials such as metal oxides, CNTs can avoid sensor poisoning and improve the long-term stability of the device. CNTFET-based gas sensors can detect gas molecules such as water vapor, NO_2_, NH_3_, H_2_, H_2_S, C_2_H_5_OH and methanol vapor, with very good detection limits and detection ranges, and their anti-noise capabilities and detection accuracy are significantly better than traditional gas sensors, and even can detect a single gas molecule [36]. Some recent works are summarized in Table 1. 

The research on carbon nanotube gas sensors began with the work published by Kong [37]. Gas-sensitivity characteristics of CNTFET to NH_3_ and NO_2_ were investigated by Tans [38]. The research found that, when exposed to NH_3_, the Fermi level in the p-type CNTFET shifts to the conduction band, which results in a decreased hole concentration and thus a decreased conductance; when exposed to NO_2_, the Fermi level shifts to the valence band, and the hole concentration and the conductance increases. In addition, the transfer curve would show a relative difference when exposed to different concentrations of the same gas.

**Table 1:** The research on CNTFET for gas detection.

Analytes	Detection Limit	Response Time	Author	References
NO_2_	200 ppm	2–10 s	Kong	[37]
NO_2_	ppb level	Not reported	Sacco	[18]
NO_2_	10 ppb	Not reported	L. Valentini	[39]
NO_2_	125 ppt	Not reported	Kumar	[40]
NH_3_	1%	1–2 min	Kong	[37]
Carbonyl Chloride	630 nm/refractive index unit	Not reported	Ghodrati	[41]
Methanol	1.3%	Not reported	Badhulika	[42]
Ethanol	5.95%	Not reported	Badhulika	[42]
Ethanol	50 ppm	Nor reported	Sean Brahim	[43]
Ethanol	1.67%	Not reported	S. J. Young	[44]
Methyl ethyl ketone	3%	Not reported	Badhulika	[42]
Nitrogen dioxide	1 ppm	Not reported	Radouane	[45]
Carbon monoxide	20 ppm	Not reported	Radouane	[45]
Ammonia	1%	0.1 s	panelF.Villalpando-Páez	[46]
Ammonia	100 ppb	15 min	Qifei Chang	[47]

**Figure 3:**
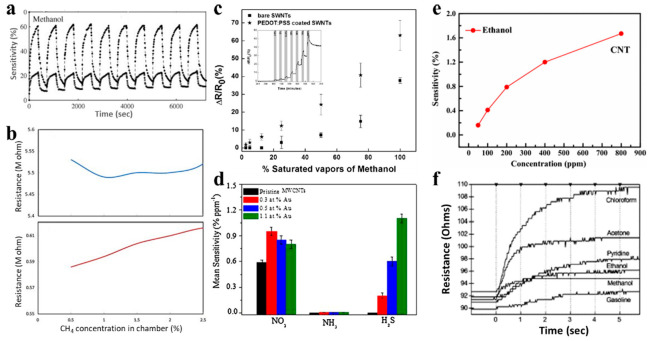
CNTFET-based gas sensor. (**a**) The response of CNTFET to methanol with (open symbols) and without (filled symbols) KMnO_4_ treatment [48]. (**b**) The response of polyaniline (PANI) (top) and multi-walled CNT (MWCNT)-PANI (bottom) membranes to methane gas [49]. (**c**) The response of SWCNTs with PEDOT:PSS coating to methanol [42]. (**d**) Selective response of CNTFET sensor functionalized with Au nanoparticles [52]. (**e**) CNTFET ethanol gas sensors functionalized with 10 nm Fe layer [44]. (**f**) Aligned CNx nanotubes sensors in response to different vapors [46].

Figure 3 shows several representative works of gas sensing with a CNTFET. Slobodian [48] reported the detection of methanol gas by using MWCNTs. The reaction of MWCNT FETs to methanol after treatment in acidic KMnO_4_ increased by about 12–46%, as shown in Figure 3a. Sattari [48] spin-coated a composite of MWCNTs and polyaniline (PANI) on glass and silicon substrates and performed sensor measurements on methane gas. The MWCNT–PANI membrane presents greatly enhanced sensitivity to methane gas compared to the pure PANI membrane, [49] as shown in Figure 3b. Badhulika [42] used SWCNTs with poly(3,4-ethylenedioxythiophene) polystyrene sulfonate (PEDOT:PSS) coating to make volatile organic compounds (VOCs) gas sensors. For saturated vapors of methanol (Figure 3c), ethanol and methyl ethyl ketone, the detection limits are 1.3%, 5.95% and 3%, respectively. Zhao [50] pointed out that NO_2_ and NH_3_ gases have strong enough interactions with CNTs (chemical adsorption), while other gases with low adsorption energy only bind to CNTs by Van der Waals force (physical adsorption). Woods [51] points out that the molecules of volatile organic compounds (VOCs) interact weakly with CNTs. The weaker interaction between VOCs and CNTs reduces the performance of their gas sensors. Therefore, in monitoring VOCs, increasing the reactivity of CNTs is a key point.

Different approaches have been studied for the modification of CNTs to improve the sensing performances, including using Au nanoparticles, certain solutions, polymers, metals, or impurities. 

CNTFET [52] gas sensors pretreated with Au nanoparticles prepared by electrophoretic deposition method have high sensitivity and selectivity to NO_2_ and H_2_S, as shown in Figure 3d. In S. J. Young’s study [44], a 10-nm thick Fe layer was sputtered followed by synthesizing CNTs, and the fabricated CNT ethanol gas sensors can reach a sensitivity of 1.67% under 800 ppm ethanol vapor concentration at room temperature, as shown in Figure 3e. Radouane [45] used MWCNTs decorated with tin oxide for sensing 1 ppm nitrogen and 20 ppm monoxide. This hybrid sensor has an excellent sensitivity and significantly eliminates the moisture cross-sensitivity. 

Impurity atoms have been incorporated into CNTs to improve their gas sensing performance, such as pyridine-like sites, boron, and nitrogen atoms. F.Villalpando-Páez [46] reported aligned CNx nanotubes sensors that are capable of sensing the toxic species because of the presence of highly reactive pyridine-like sites on the tube surface which can strongly bind to ammonia, acetone and OH groups (Figure 3f) and further change their density of states. This type of sensor is both responsive and reusable at the same time. To overcome the reliability problem owing to the weak van der Waals interaction between the SWCNTs and the doped materials, Shu Peng [53] reports a concept of a brand new type of CNT-based gas sensor by doping the impurity atoms (such as boron, nitrogen atoms) into SWCNTs.

### 4.2. H_2_O_2_ Detection

**Figure 4:**
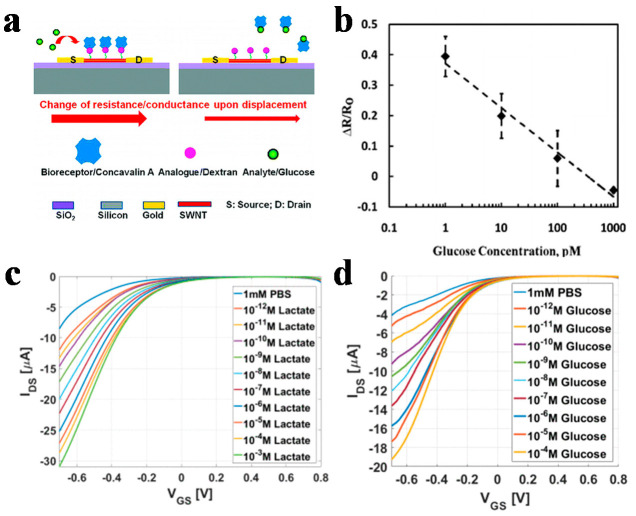
CNTFET-based H_2_O_2_ sensor. (**a**) Schematic of a CNTFET for glucose sensing via the detection of H_2_O_2_ and (**b**) its relative resistance change in response to different glucose concentrations [54]. The change of current of CNTFETs in response to (**c**) different lactate and (**d**) glucose concentrations via enzymatic reactions with H_2_O_2_ as a by-product [55].

H_2_O_2_ is a by-product of most enzyme-catalyzed reactions and is closely related to the occurrence of many metabolites in the body, such as glucose, lactate, cholesterol. Thus, the detection of H_2_O_2_ can be correlated to the concentration of these metabolites through specific enzymatic reactions. Figure 4a shows the schematic of a CNTFET sensor for H_2_O_2_ with the bioreceptor of concavalin A, in which H_2_O_2_ is intermediate of glucose reaction, and the concentration of H_2_O_2_ and thus the concentration of glucose can be detected by CNTFET sensor. [54] The calibrated curve in Figure 4b shows a good linear relationship between the relative resistance change of the sensor and the glucose concentration, which is tested in human plasma, revealing the potential application of the sensor for blood glucose measurements. Saumya [55] used CNTFET sensors to detect lactate (Figure 4c) and glucose (Figure 4d) via the enzymatic reaction catalyzed by glucose oxidase and lactate oxidase, respectively, with H_2_O_2_ as a product of the reaction. This method possesses a good monitoring sensitivity, and a decent detection limit of pico molar (pM) levels.

## 5. CNTFET-Based Biological Sensors

Label-free biosensors are very attractive due to their simple procedure, high sensitivity, rapid detection, easy of miniaturization and integration. The CNT with a 1-D nanostructure has shown a strong sensitivity to the surface adsorption of many chemicals and biomolecules. This enables the CNT to be an ideal material for constructing label-free biosensors to detect proteins [56,57], nucleic acids, cells, and viruses [58,59,60].

### 5.1. Protein Detection 

**Figure 5:**
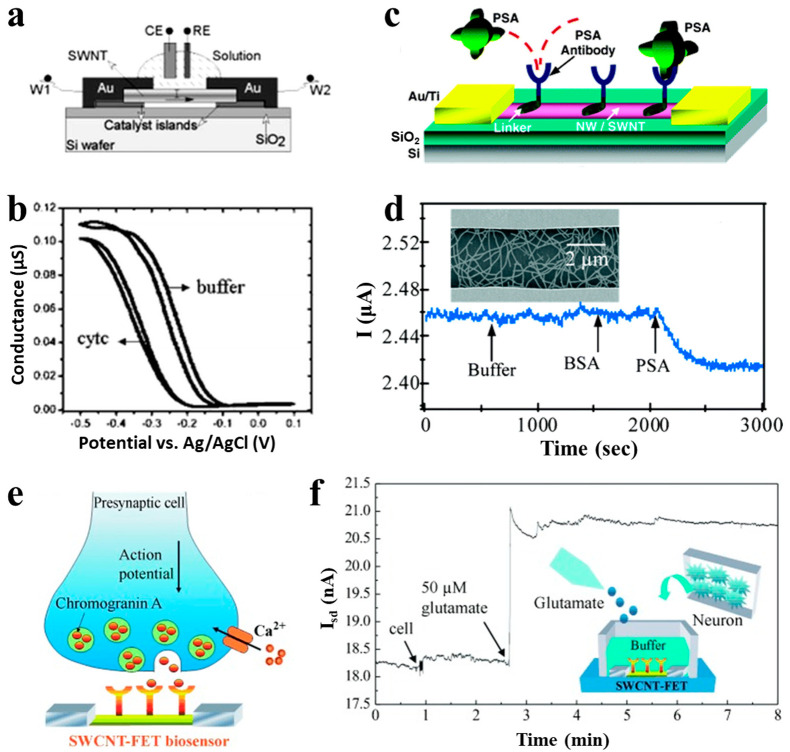
CNTFET-based sensors for protein detection. (**a**) Schematic illustration of a SWCNT device for cytochrome c detection [61]. (**b**) Conductance of the CNTFET as a function of the electrochemical potential in phosphate buffer and 200 µM cytochrome c in buffer. (**c**) Schematic device diagram and (**d**) current change of CNT nanosensors for prostate-specific antigen (PSA) detection [62]. (**e**) Schematic illustration of chromogranin (CgA) released from neurons and (**f**) its detection by monitoring the current change of a CgA antibody-modified CNTFET [63].

CNTs can be functionalized with specific antibodies to detect different proteins. When proteins are bound onto CNTs by receptors on the surface, it leads to a change of source and drain current and voltage. Some recent works are summarized in Table 2. Boussaad [61] detected the non-covalent adsorption amount of cytochrome c in situ by monitoring the conductance change in semiconductor CNTs. The basic structure of a SWCNT device is shown in Figure 5a. The detection sensitivity is high, up to 20 protein molecules/carbon tubes, as shown in Figure 5b. The possible reason for the change is that the positively-charged cytochrome c reduces the electronic load of the p-type semiconductor CNTs, resulting in a decrease in conductance. Figure 5c shows the illustration of the CNTFET sensor for prostate-specific antigen (PSA) detection [62]. PSA monoclonal antibody was immobilized on the SWCNT surface and PSA can be bound to the antibody for recognition. When the device was exposed to 1.4-nM PSA, the current was changed about 2% as shown in Figure 5d. Wang [63] used an antibody-functionalized CNTFET biosensor to detect in situ chromogranins (CgA) released from neurons, as shown in Figure 5e,f, where small current increase was observed when neurons were attached onto the CgA antibody-modified SWCNT FET. Marcin [64] fabricated sorted CNT networks with a nanobody receptor for protein detection up to 1 pM.

### 5.2. Cell Detection

The CNTs can be functionalized with the specific antibodies to detect different cells, such as bacterial, pathogenic yeast, or mammalian cells. The living cell could also be absorbed on the CNT surface because of physical or chemical reasons, which could lead to the change of transfer curve, make it possible for detection. 

**Figure 6:**
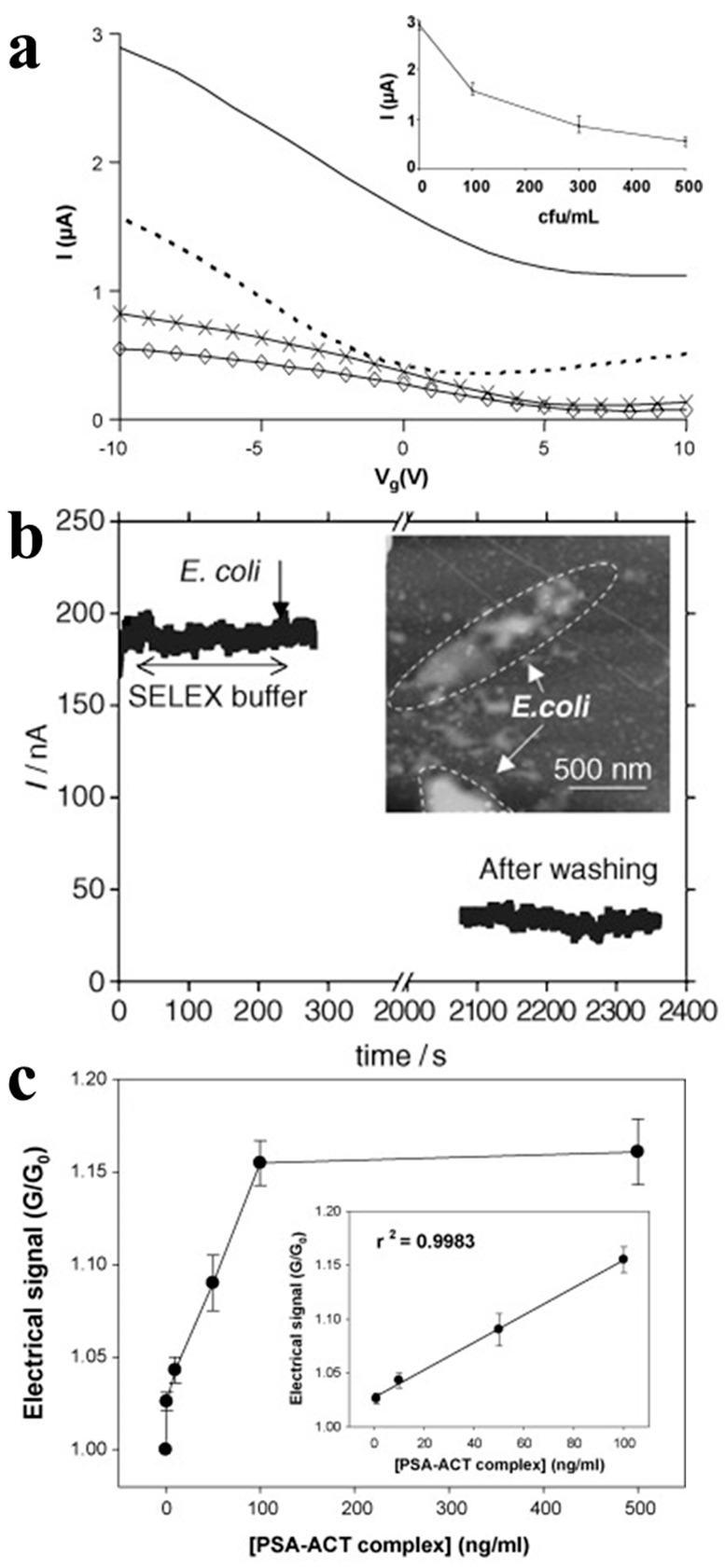
CNTFET-based sensors for cell detection. (**a**) From top to bottom, transfer curves before and after exposure to Salmonella with concentrations of 100 cfu/mL, 300 cfu/mL and 500 cfu/mL, respectively [73]. (**b**) Current change of the CNTFET for Escherichia coli detection [74]. (**c**) Prostate-specific antigen PSA-ACT complex detection on the CNTFETs modified with a 1:3 linker to spacer ratio [75].

Villamizar [73] reported a study using Salmonella antibodies to functionalize CNTFETs for Salmonella detection at a minimum concentration of 100 colony-forming units (cfu)/mL, as shown in Figure 6a. In addition, a similar method has been used to construct biosensors by functionalizing CNTFETs with Escherichia coli aptamer for Escherichia coli detection [74]. The conductivity of the device is reduced by more than 50% after the Escherichia coli is captured (Figure 6b). 

Kim [75] proposed a label-free protein biosensor based on a functionalized CNTFET for detecting the prostate cancer marker, PSA-α_1_-antichymotrypsin (PSA-ACT complex), as shown in Figure 6c. They functionalized the CNTFET with a 1:3 ratio of linker-to-spacer. Owing to addition of spacers on the CNT surface, this approach could widen the distance between the receptors. Thus, the negatively charged proteins can approach the channel within the distance of the Debye length to further affect the conductance of the CNTFET more easily. This results in a sensitive detection of 1.0 ng/mL. 

### 5.3. Nucleic Acid Detection

Nucleic acid analytes, such as DNA and RNA, can be effectively combined with CNTFET biosensors. The detection of DNA by sequence-specific hybridization is a common detection strategy to ensure the specificity of biosensors. The FET biosensor can functionalize complementary DNA/RNA/peptide nucleic acid (PNA) strands to the sensor surface, so that the complementary binding target strand can produce the specific binding to ensure its selectivity and produce a detectable electrical response. Some recent work has been summarized in Table 3. 

**Figure 7:**
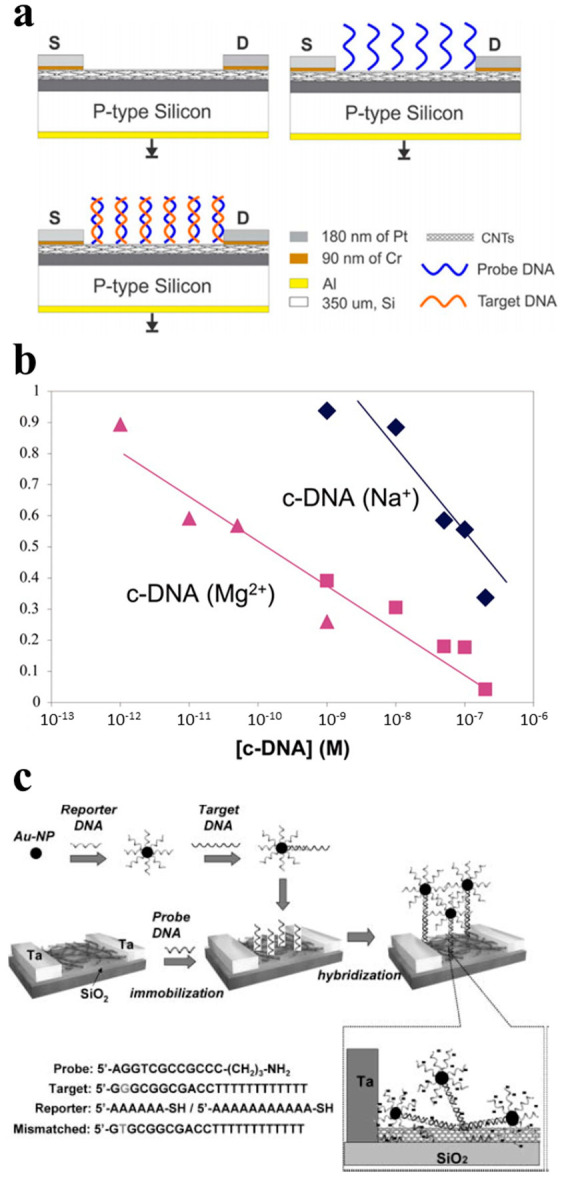
CNTFET-based sensors for DNA detection. (**a**) Working principle of CNTFET-based DNA sensors [91]. (**b**) Normalized conductance as a function of target DNA concentrations [87]. (**c**) Schematic illustration of DNA detection with reporter DNA conjugated with Au nanoparticles [88].

As shown in Figure 7a, the sensing of DNA relies on the binding of DNA double-strand which has an effect on the CNTFET transfer curve [91]. The concentration of DNA will be detected by the shift degree of the transfer curves. Star [87] reported a CNT network FET for the detection of a specific DNA sequence.

A sequence. This kind of sensor showed the ability to recognize the target DNA sequences by immobilizing the synthetic oligonucleotides. It was further approved that ssDNA was successfully immobilized and hybridized with the target subsequent DNA by utilizing fluorescence-labeled oligonucleotides. The sensor had a detection limit of 1 nM (Figure 7b), and was both highly sensitive and low-cost. Dong [88] further improved the CNTFET DNA sensor by labeling a reporter DNA probe with Au nanoparticles in the hybridization step, and the detection process is illustrated in Figure 7c. MAEHASHI [89] demonstrated a CNTFET-based DNA sensor which covalently immobilized amino modified PNA oligonucleotides at 5′ onto the Au surface of the back gate. The sensor can detect DNA as low as 6.89 fM.

### 5.4. Virus Detection

**Figure 8:**
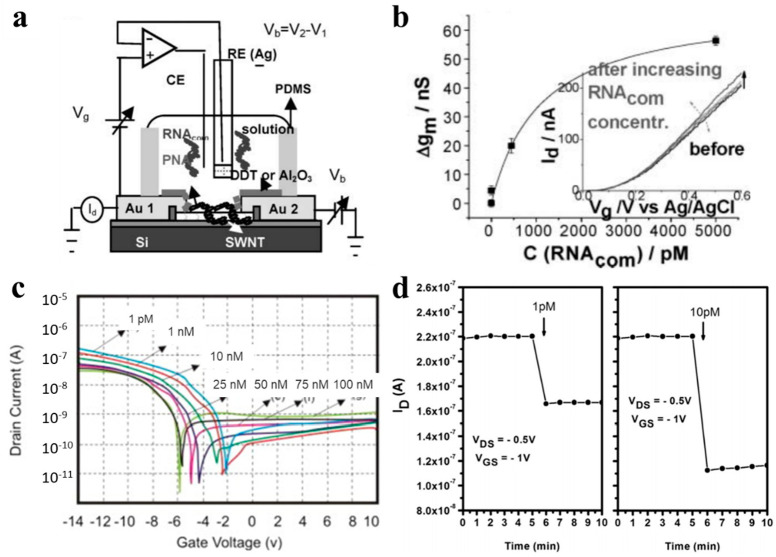
CNTFET-based sensors for virus detection. (**a**) The experimental setup and (**b**) transconductance change vs. RNA concentrations of the hepatitis c virus (HCV) sensor [92]. (**c**) Detection of H5N1 at different concentrations [93]. (**d**) Response to influenza type A virus DNA within 1 min [91].

CNTFET-based biosensors can be used for the monitoring of a variety of viruses. The virus sensing is usually based on detecting the DNA of the virus through the immobilization of DNA or PNA, or directly detecting the virus through the immobilization of the antibody or peptide. Some recent works are summarized in Table 4. Dastagir [92] constructed an FET sensor using functional CNT as the channel material to monitor the sequence of the hepatitis c virus (HCV) (Figure 8a). They used PNA, which possesses high affinity and stability for RNA hybridization, to perform CNT functionalization, and a sensor that could carry on an unlabeled detection with a detection limit of 0.5 pM, as shown in Figure 8b. 

Thu [93] reports on a CNTFET for the selective detection of H5N1. In this sensor, the CNT network acts as a conductor channel, producing signal changes when the virus binds to specific receptors on the CNT surface. The specific DNA sequence was a receptor bound to the H5N1 virus, with a detection limit of up to 1.25 pM and a sensitivity of 0.28 nM/nA, as shown in Figure 8c. Tran [91] reported a CNTFET-based sensor for the detection of influenza type A virus DNA. They used an immobilized single DNA strand on the CNT network as the probe which would hybridize with the analyte DNA to further alter the ion concentration on the surface. As the ion concentration changes, the transfer curve of the CNTFET changes. This sensor has a detection limit of 1 pM within a linear detection range from 1 pM to 10 nM and also shows superior responsiveness in less than one minute, the response time is shown in Figure 8d. Fatin [94] demonstrates a MWCNT-based sensor for HIV-1 virus which utilized a split RNA aptamer as the detection probe. The sensor has a detection limit of 600 pM. 

**Table 4:** The research on CNTFET for virus detection.

Analytes	Detection Limit	Sensitivity	Author	References
H5N1 virus	1.25 pM	0.28 nM/nA	Vu	[93]
Influenza A virus	10 μM	Not reported	Thi	[87]
Avian influenza virus	1 EID_50_ (50% embryo infectious doses)/mL	Not reported	Yin-Ting Yeh	[95]
H1N1	180 TCID_50_ (50% tissue culture infective dose)/mL	Not reported	Dongjin Lee	[96]
Hepatitis C virus	pM level	Not reported	Tawab Dastagir	[92]
Dengue virus	2 ng mL^−1^	Not reported	Naimish P. Sardesai	[68]
M13-bacteriophage	0.5 pM	Not reported	HS Mandal	[97]
Dengue virus	0.1 µg mL^−1^	Not reported	Mízia M. S. Silva	[98]
Hepatitis B	0.03 ng mL^−1^	Not reported	Diego G.A.Cabral	[99]
Hepatitis B	<1 attomole	Not reported	ShunWang	[6]
SARS-CoV-2	35 mg/L	Not reported	Rebecca L. Pinals	[100]
Hepatitis C	0.7 fM	Not reported	B Zribi	[85]
Papilloma virus	<1 attomole	Not reported	ShunWang	[82]
Human papilloma virus	130 μA/V	Not reported	Gopinath	[101]

## 6. Conclusions

The CNTs have shown the extraordinary electrical and sensing properties, and we here review the recent progress of the CNTFET-based chemical and biological sensors. The CNTs generally go through covalent or non-covalent surface functionalization, in order to generate sufficient or selective sensing signals. The configurations include the general FET structure and electrolyte-gated FETs. The CNTFET-based sensors have been applied for the effective chemical sensing of various gases or H_2_O_2_. The bioreceptor functionalization of the CNTFET enables the construction of various biological sensors for detecting a variety of proteins, cells, nucleic acids, and viruses. Due to the nanoscale channel with a large surface to volume ratio and an atomically thin body to provide ideal electrostatic control, a small change of the surrounding environment can significantly modify CNTFETs’ electrical characteristics, and, thus, a small concentration of the target analyte that can bind to the surface of the channel can result in a detectable change of the electrical signal. So, these sensors generally exhibit a highly sensitive detection of the target analyte. We expect the CNT transistor-based sensors to deepen the fundamental understanding of the interaction of the analyte with the CNTFET, as well as explore a wide range of applications in healthcare, environmental, and food. Although the progress for the CNTFET-based chemical and biological sensors is promising, the field still faces the critical challenges for practical applications, and these include (1) the unstable electrical performance of the CNTFET over time; (2) the fluctuation of the sensing performance by small variation of the surroundings, such as the buffer condition; and (3) the non-linear calibration curve between the electrical signal and the concentration of the analyte. These issues hamper the realization of portable and miniaturized systems using FET transduction and significant lowering of the manufacturing cost will also be critical to allow CNTFET-based sensors to be marketed. 

## Figures and Tables

**Table 2:** The research on CNTFET for protein detection.

Analytes	Detection Limit	Sensitivity	Author	References
Dopamine	0.062 μM	Not reported	Haiyan Cheng	[65]
Dopamine	15 nM	Not reported	Jinyan Cheng	[66]
Dopamine	0.87 nM	Not reported	Qitong Huang	[67]
Specific protein detection	0.1 pM	Not reported	Marcin	[64]
Non-structural protein 1 of the dengue virus	2 ng/mL	Not reported	sAna Carolina M.S.Dias	[68]
Prostate specific antigen	1 pg/mL	Not reported	Naimish P. Sardesai	[69]
IL-6	0.25 pg/ mL	Not reported	Naimish P. Sardesai	[69]
Hexahistidine-tagged capture proteins	10 pM	600 s	Jin-Ho Ahn	[70]
Pig serum albumin	2.06 µmol/L	Not reported	Atsuhiko Kojima	[71]
Urokinase plasminogen activator	25 nM	Not reported	Ryan M. Williams	[72]

**Table 3:** The research on CNTFET for Nucleic acid-based analyte detection.

Analytes	Receptors	Detection Limit	Author	References
12-mer ssRNA	12-mer PNA	Not Reported	Martínez	[76]
10-mer ssDNA	10-mer ssDNA probe	Single molecule	Sorgenfrei	[77]
microRNA-122a	p19 protein	1 aM	Ramnani	[78]
DNA	Amino-functionalized probe DNA	Attomolar level	Tetiana Kurkina	[79]
ssDNA	ssDNA probe	2 nM	Stine	[80]
ssDNA	Peptide nucleic acid probe	100 fM	Cai	[81]
ssDNA	ssDNA probe	<1 attomole.	ShunWang	[82]
ssDNA	ssDNA probe	<1 attomole.	ShunWang	[82]
ssDNA	ssDNA probe	2.4 nM	Yin	[83]
ssDNA	ssDNA probe	0.5 nM	Phuong DinhTam	[84]
ssDNA	ssDNA probe	0.7 fM	B Zribi	[85]
ssDNA	Amine-modified DNA detection probe	0.1 nM	Wanwei Qiu	[86]
ssDNA	ssDNA	1 pM	Alexander Star	[87]
ssDNA	ssDNA	100 fM	Xiaochen Dong	[88]
ssDNA	Amino modified PNA	6.8 fM	Kenzo Maehashi	[89]
ssDNA	Peptide nucleic acid probe	10 fM	Zheng	[90]

## Data Availability

Not Applicable.
